# Molecular cloning and sequence analysis of novel cytochrome P450 cDNA fragments from *Dastarcus helophoroides*

**DOI:** 10.1093/jis/14.1.28

**Published:** 2014-01-01

**Authors:** Hai-Dong Wang, Fei-Fei Li, Cai He, Jun Cui, Wang Song, Meng-Lou Li

**Affiliations:** 1 Laboratory of Forestry Pests Biological Control, College of Forestry, Northwest A&F University, Yangling, Shaanxi, 712100, P. R China; 2 College of Science, Northwest A&F University, Yangling, Shaanxi, 712100, P. R China; 3 Wuwei Academy of Forestry Sciences, Wuwei, Gansu 733000, P.R. China; § These authors contributed equally to this work

**Keywords:** homologous analysis, CYP6 fragments

## Abstract

The predatory beetle *Dastarcus helophoroides* (Fairmaire) (Coleoptera: Bothrideridae) is a natural enemy of many longhorned beetles and is mainly distributed in both China and Japan. To date, no research on *D. helophoroides* P450 enzymes has been reported. In our study, for the better understanding of P450 enzymes in *D. helophoroides* , 100 novel cDNA fragments encoding cytochrome P450 were amplified from the total RNA of adult *D. helophoroides* abdomens using five pairs of degenerate primers designed according to the conserved amino acid sequences of the CYP6 family genes in insects through RT-PCR. The obtained nucleotide sequences were 250 bp, 270 bp, and 420 bp in length depending on different primers. Ninety-six fragments were determined to represent CYP6 genes, mainly from CYP6BK, CYP6BQ, and CYP6BR subfamilies, and four fragments were determined to represent CYP9 genes. Twenty-two fragments, submitted to GenBank, were selected for further homologous analysis, which revealed that some fragments of different sizes might be parts of the same P450 gene.

## Introduction

Cytochrome P450 monooxygenases are one of the most important enzyme systems found in most organisms. Various P450 genes obtained from animals, plants, and microorganisms have been classified into numerous families ( Feyereisen 2006 ). P450 genes bind molecular oxygen and receive electrons from NADPH to introduce an oxygen atom to the substrate ( Nelson 1993 ). In insects, the diverse functions of P450 enzymes range from the synthesis and degradation of ecdysteroids and juvenile hormones to the metabolism of xenobiotics ( Nelson 2006 ). Though P450 monooxygenases can be detected in virtually all tissues, the highest monooxygenases activities are usually associated with the midgut, fat bodies, and Malpighian tubules (Feyereisen 2006).

Insect CYP superfamily P450 genes, mostly beloning to CYP4, CYP6, CYP9, CYP12, CYP18, CYP28, and CYP301-318 families, have frequently been involved in the detoxification processes that allow tolerance of insects to insecticides ( Scott et al. 1998 ; Feyereisen 2005 ). As increasing numbers of P450s were isolated from different species, their functions in the metabolism of endogenous compounds were constantly revealed. For instance, CYP6L1 was highly and exclusively expressed in the testes and accessory glands of male adult German cockroaches, *Blattella germanica* , which strongly indicated that CYP6L1 played a role in reproduction ( Wen and Scott 2001 ). CYP6BQ13v2, an allele of CYP6BQ13, isolated from the red flour beetle, *Tribolium castaneum* , was expressed in all life stages and was associated with the synthesis of ecdysteroids; further quantitive analysis revealed that the CYP6BQ13v2 expression levels appeared in the order of 1st instar larvae, followed by the 4th instar larvae, 7th instar larvae, and pupae from high to low ( Xu et al. 2009 ). CYP6CM1, an age-specific gene isolated from the whitefly, *Bemisia tabaci* , was proved to play a role in the development process ( Jones et al. 2011 ). It was estimated that nearly 65% of insect P450s are involved in the metabolism of endogenous compounds ( Scott 2008 ).

*Dastarcus helophoroides* (Fairmaire) (Coleoptera: Bothrideridae) is a natural enemy of *Anoplophora glabripennis* , *Monochamus alternatus* , *Batocera horsfieldi* , *Massicus raddei* , and many other longhorned beetles ( Wei and Yang 2009 ). These parasitic beetles are distributed in both China and Japan ( Qin and Gao 1988 ; Miura et al. 2003 ; Urano 2003 ). *D. helophoroides* larvae are ectoparasitoids of late instar larvae, pupae, and young adults of several longhorned beetle species, which makes them a potential biological control agent for pest management ( Wang et al. 1996 ; Zhang and Yang 2006 ; Li et al. 2007 ). The adult *D. helophoroides* has a relatively longer lifespan than other insects, potentially living eight or more years in laboratory. Though the morphology and physiology of *D. helophoroides* has been widely reported, its aging mechanism is unknown ( Lei et al. 2003 ). To date, there is no study or report on *D. helophoroides* P450 enzymes. Considering the wide range of functions in insects, it is reasonable to speculate that P450s might play an important role in the aging process of *D. helophoroides* . Also, the isolation and characterization of insect P450s is the critical first step towards the understanding of the function of P450s in certain metabolic process ( Scott 1999 ; Feyereisen 2005 ). In the present paper, a cluster of P450 cDNA fragments were amplified from the total RNA of adult *D. helophoroides* abdomens using RT-PCR. Furthermore, homologous analysis indicated that these fragments are all new P450 cDNA sequences, belonging to CYP6 and CYP9 families.

## Materials and Methods

### Adult insects

The *D. helophoroides* adults were provided by the Laboratory of Forestry Pests Biological Control, College of Forestry, Northwest Agriculture and Forestry University, Yangling, Shaanxi, P. R China. The adults were maintained and fed at 22 ± 1°C and 80% RH under a 16:8 L:D photoperiod. The major components of the diet were silkworm pupa powder, sugar, yolk, and agar. The diet was prepared as described by Lei et al. (2005) .

### Isolation of total RNA, and synthesis of the first strand of cDNA

Total RNA for amplification of P450 cDNA fragments was isolated from the abdomen of adult *D. helophoroides* using RNAiso Plus (Takara Bio, www.takarabio.com ). Five adult individuals were dissected to remove their abdomens, which then were ground with liquid nitrogen until becoming a powder. The powder was quickly transfered into a 1.5mL centrifuge tube and homogenized with 1mL RNAiso Plus (Takara). The process of total RNA extraction and purification was carried out according to the manufacturer’s instructions. The total RNA (A260/A280 = 1.865) was dissoved in 20 µL DEPC treated H2O and stored at -80°C. The first strand of cDNA was synthesized using 2 µg of the total RNA using a TIANScript First Strand cDNA Synthesis Kit (Tiangen Biotech, www.tiangen.com ) following the manufacturer’s instructions. The reaction mixture was stored at -20°C.

### Degenerate primers and amplification of the cDNA fragments

Four pairs of degenerate primers (dpE, dpF, dpG, and dpH) were designed according to the conserved amino acid sequences of insect CYP6 genes, and another pair of degenerate primers (dpA) reported by Kasai et al. (1998) were taken for comparison ( Table 1 ). The amplification was performed with S1000 PCR Thermal Cycler (Bio-Rad, www.bio-rad.com ) using 2X ES Master mix Taq polymerase (CWBIO, www.cwbiotech.com ). The total reaction volume was 25 µL, including 1 µL cDNA, 1 uL 10 µM/L forward and reverse primer respectively, 12.5 µL 2X ES Master mix Taq polymerase, and 9.5 µL ddH2O. The PCR program was conducted under the following conditions: an initial denaturation at 94°C for 1 min followed by 30 cycles of 94°C for 30 sec, 45°C for 30 sec, and 72°C for 1 min, and a final extension at 72°C for 10 min. The PCR products were then separated by 1.5% agarose gel electrophoresis and stained with ethidium bromide (EB). The bands of the expected size (250 bp, 270 bp, and 420 bp) were excised and recovered using Universial DNA Purification Kit (Tiangen Biotech).

**Table 1.**
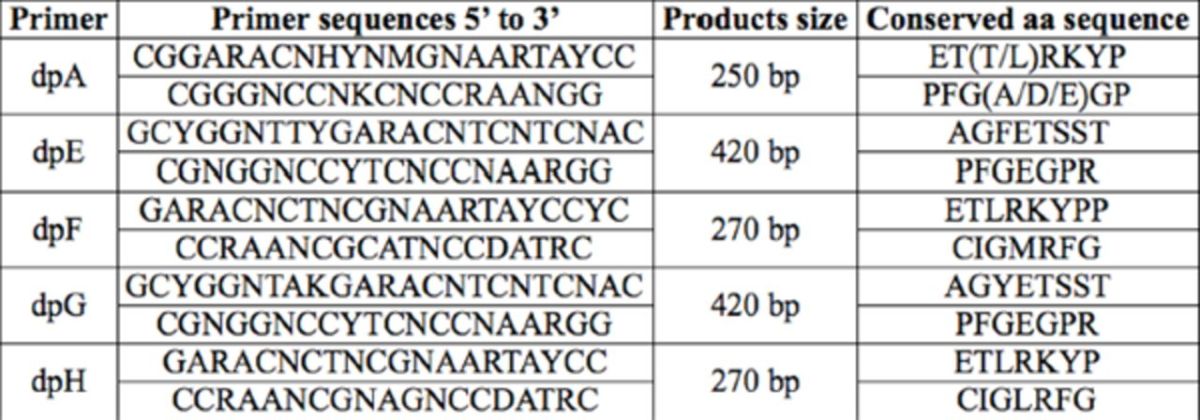
Primers used in RT-PCR

Primer dpA was quoted from Kasai (1998) . Primers dpE, dpF, dpG, and dpH were original.

### Cloning and sequencing of cDNA fragments

The purified fragments were cloned using pUCm-T Vector Cloning kit (Sangon Biotech, www.sangon.com ). After transformation into SK2301 competent cells (Takara), the DNA inserts of the recombinant clones were amplified by PCR with the five degenerate primers used above, and sequenced in both directions.

### Bioinformatic analysis

The cDNA sequences were compared with the other similar P450 genes registered in Gen-Bank using Blastx (searching protein database using a translated nucleotide query) ( http://blast.ncbi.nlm.nih.gov ). The alignment of the cDNA sequences and deduced amino acid sequences were conducted by DNAMAN 6.0 (Lynnon, www.lynnon.com ) and Clustalx 2.0 ( www.clustal.org ). The deduced amino acid identity matrix was derived by MatGAT 2.01. The printing and shading of the Multiple-Alignment was carried out by BOXSHAD3.21 ( www.ch.embnet.org/software/BOX_form.html ). The phylogenetic tree was constructed by MEGA 5.0 ( www.megasoftware.net ) ( Kumar et al. 2008 ) using the neighbor-joining method.

## Results

### cDNA amplification and cloning

**Figure 1.**
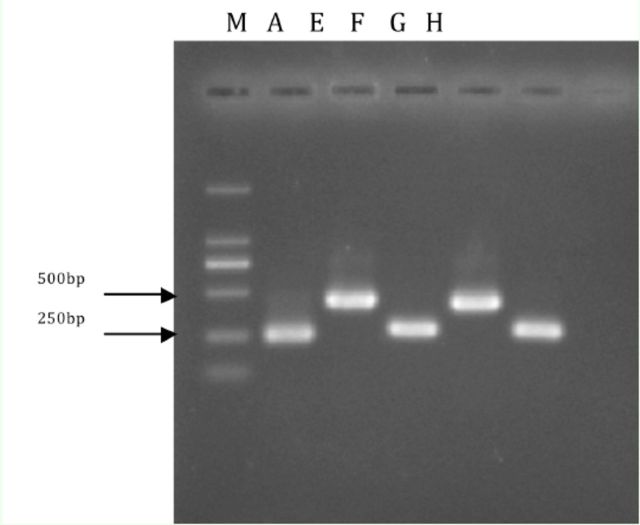
PCR products by different primers. Products A, E, F, G, and H were derived by primers dpA, dpE, dpF, dpG, and dpH, respectively. Product lengths (bp) from left to right are: 250, 420, 270, 420, 270. M = marker. High quality figures are available online.

The sizes of the PCR products were 250 bp,270 bp, and 420 bp ( Figure 1 ), depending on different primers. The five purified PCR products (A, E, F, G, H) were then cloned using pUCm-T Vector Cloning kit.

### cDNA sequencing and characterization

Twenty-five positive clones from each of the products A, E, F, G, and H (n = 125 total clones) were sequenced. In order to balance the sequence number of each product, 20 sequences that were determined to represent P450 genes were selected for each product. A Blastx search of GenBank using the total 100 nucleotide sequences as queries revealed various levels of homology with CYP6 and CYP9 genes from other organisms. Ninety-six sequences were determined to represent CYP6 genes, and four sequences were determined to represent CYP9 genes. All of the CYP6 sequences had significant homology to genes from either *Tribolium castaneum* (CYP6BK17, CYP6BK5, CYP6BQ13, CYP6BR3) or *Pediculus humanus corporis* . Nucleotide sequences that shared a ≧96% nucleotide identity in the same product cluster were classified into a same contig. The 100 CYP6 and CYP9 sequences were aligned into 17 distinct contigs (CP-1 to CP-17) that represented six unique P450 genes ( Table 2 , Table 3 ).

**Table 2.**
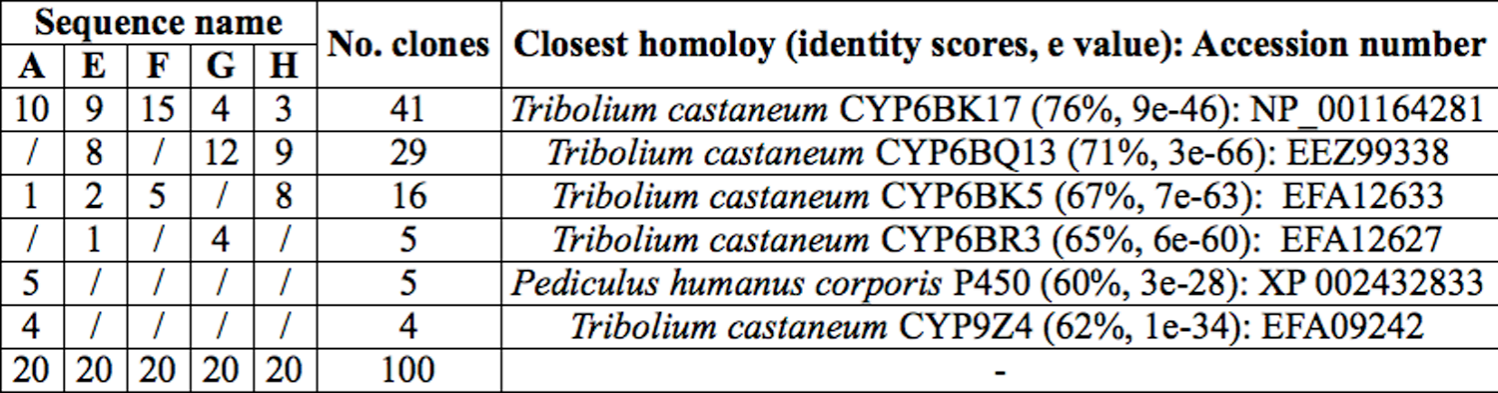
Sequence summary of 100 clones.

**Table 3.**
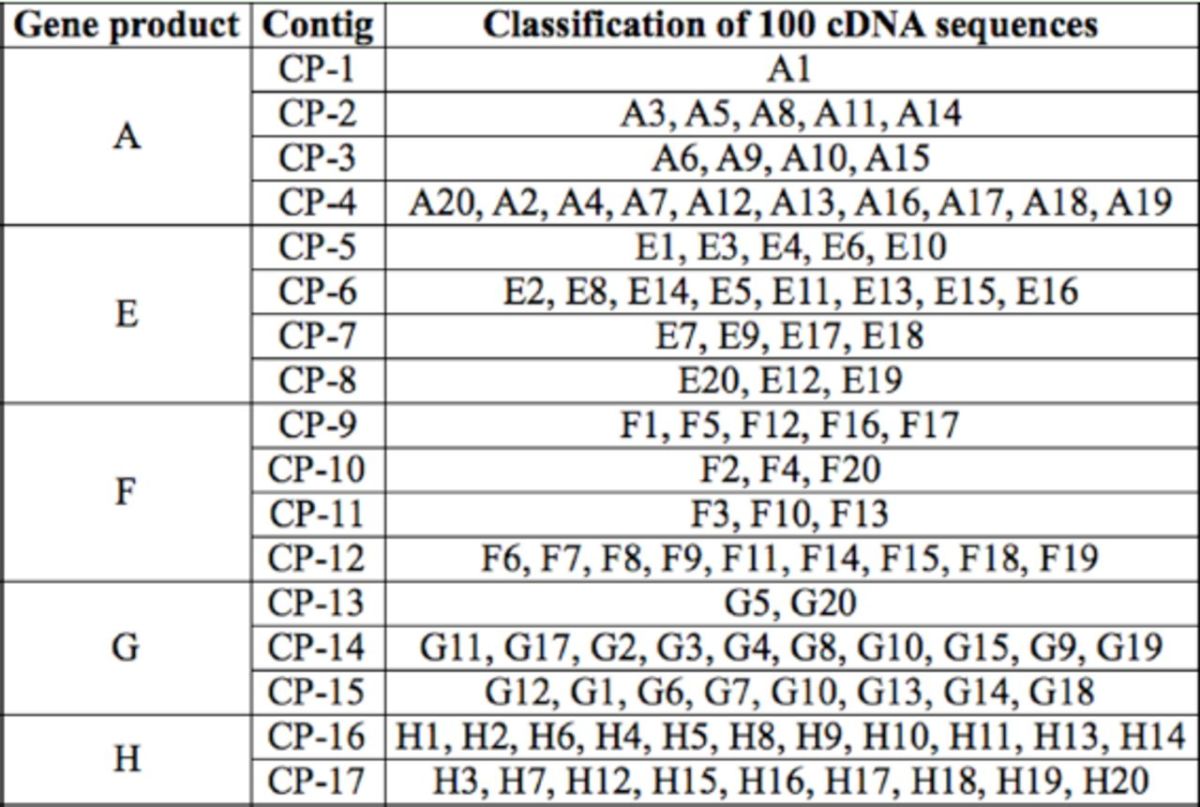
Classification of 100 cDNA sequences.

Twenty-two sequences were selected from the 17 contigs as candidates and divided into two groups (I and II) on the basis of sequence length for further analysis. All 22 fragments were submitted to GenBank, and the accession numbers were obtained. According to the best matches of the 22 novel sequences ( Table 4 ), genes from CYP6BK and CYP6BQ subfamilies accounted for nearly 80% of total genes, and the other two subfamilies (CYP6BR, CYP9Z) accounted for the remaining 20%. Table 4 shows that among the 22 sequences,eight were homologous to CYP6BK17 ( *T.castaneum* ), five were homologous to CYP6BK5 ( *T. castaneum* ), four were homologous to CYP6BQ13 ( *T. castaneum* ), two were homologous to CYP6BR3 ( *T. castaneum* ), two were homologous to P450 ( *Pediculus humanus corporis* ), and one was homologous to CYP9Z4 ( *T. castaneum* ). Furthermore, product A had a relatively higher sequence diversity than the others, which corresponded to its high degeneracy.

**Table 4.**
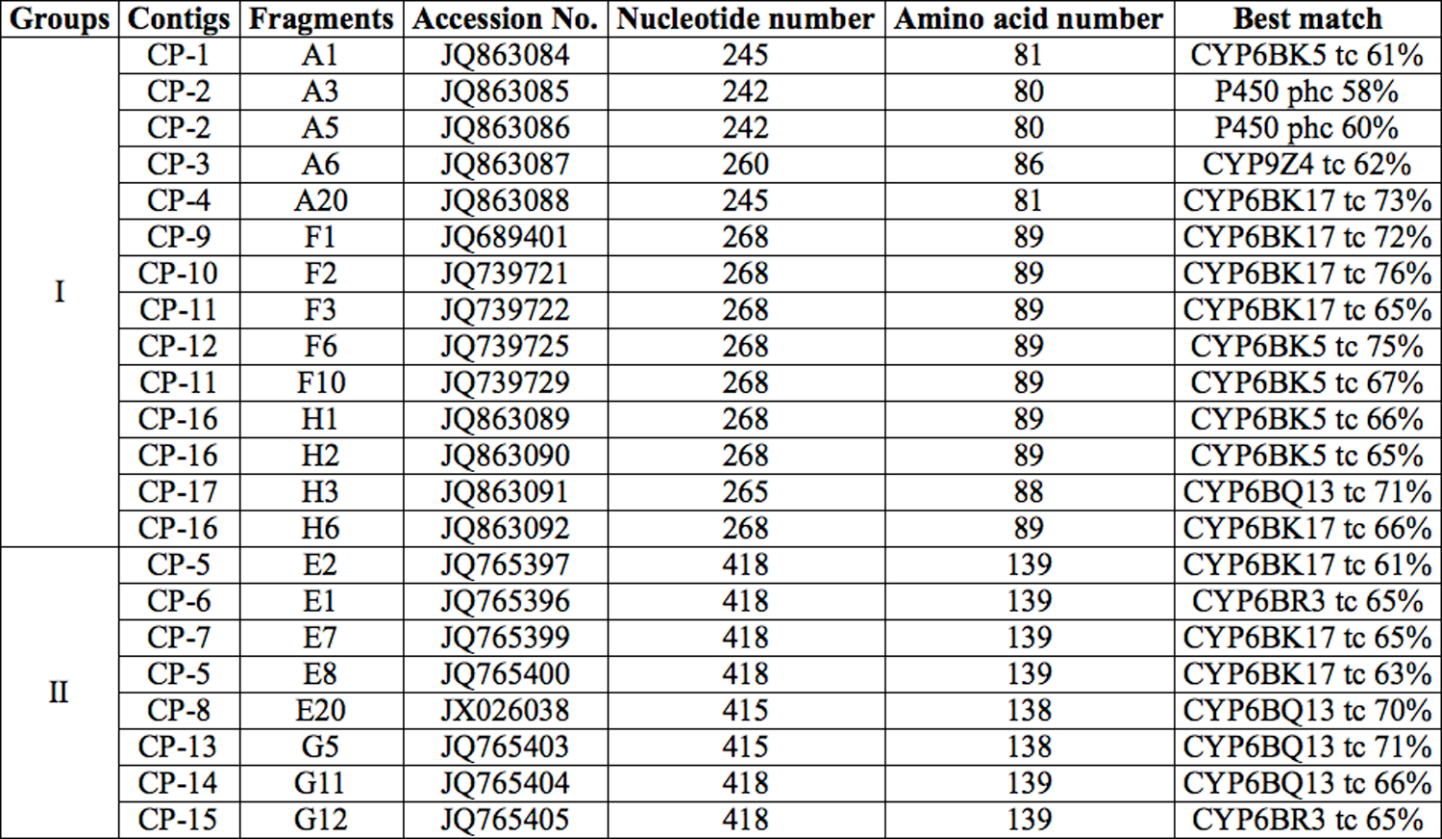
The best matches of the selected 22 P450 cDNA fragments.

tc: *Tribolium castaneum* , phc: *Pediculus humanus corporis*

### Deduced amino acid and homologous analysis

**Figure 2.**
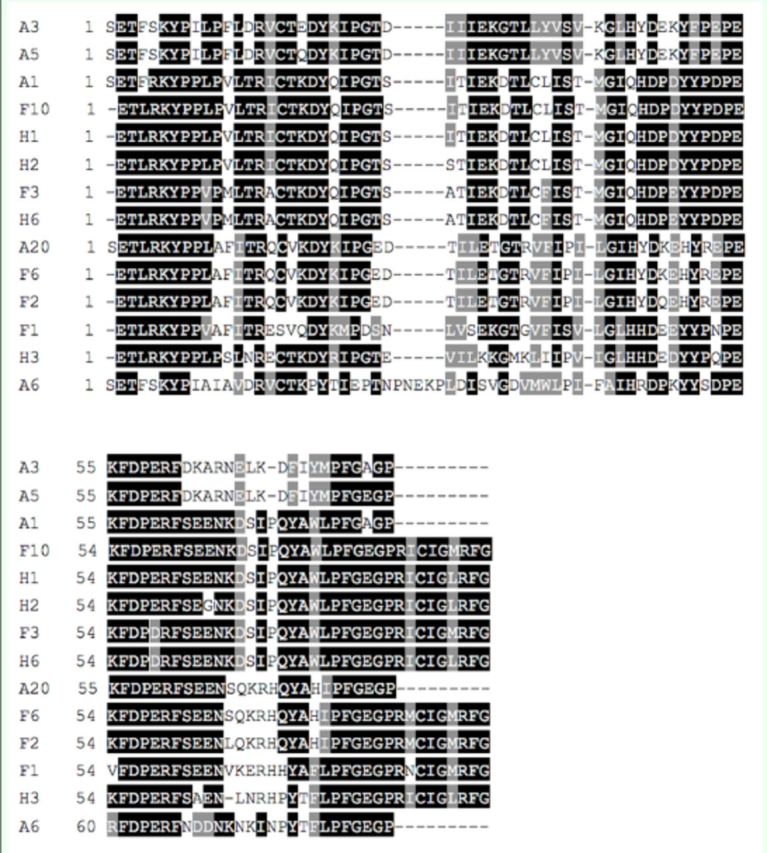
Deduced amino acid alignments of products A, F and H. High quality figures are available online.

**Figure 3.**
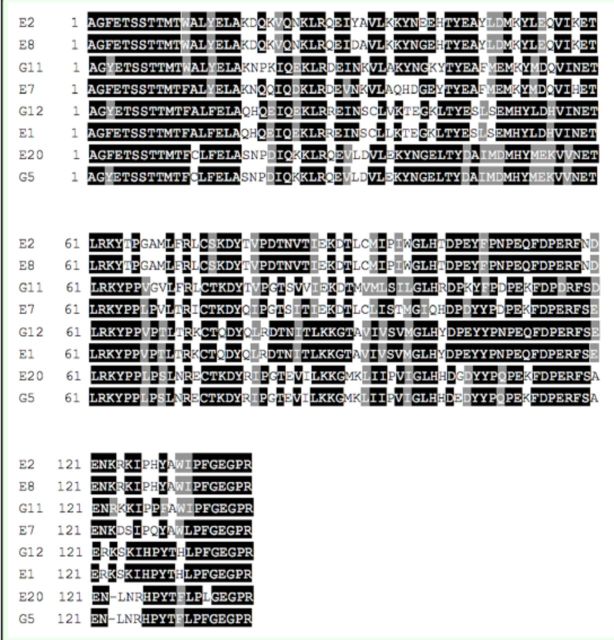
Deduced amino acid alignments of products E and G. High quality figures are available online.

The deduced amino acid identity of the 22 sequences varied from 38.4 to 100% ( Table 5 ). Sequences from the same contig shared a high amino acid identity, such as A3 and A5, F2 and F6, H1 and H2, and E2 and E8 ( Table 5 ). According to Figure 2 and Figure 3 , the heme-binding sequence motif FxxGxxxCxG (residue 77–86; 135–140) that is universal among P450 enzymes was found in all the 22 deduced amino acid sequences. As is shown in Figure 2 , with the exception of A1, A3, A5, and A6, the primer corresponding motif ETLRKYPP (residue 2–9) was found in the upstream of the other 11 deduced amino sequences. Moreover, the characteristic P450 motif PERF (residue 58–61) was found in all the other 12 deduced amino acid sequences except H3 and F6. In addition, compared with the other 13 deduced amino acid sequences, A6 had a particular sequence PNEKP (residue 28–32), which may be one of the features to distinguish CYP6 and CYP9 families. Judging from Figure 3 , the primer corresponding motif AG(Y/F)ETSST (residue 1–8) was found in all eight deduced amino acid sequences. Furthermore, the particular P450 sequence motifs ExxR (residue 59–62) and PERF (residue 115–118) were also found.

**Table 5.**
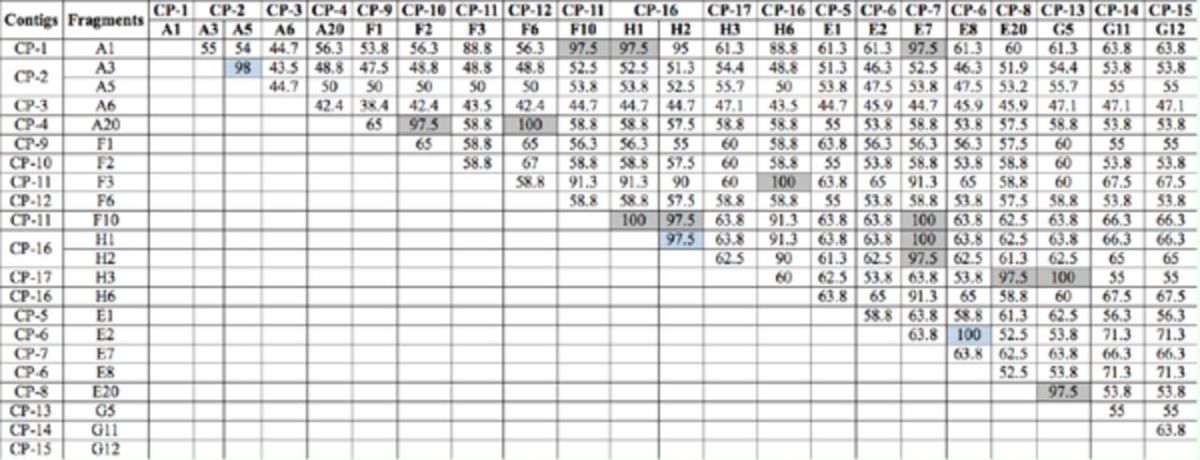
Deduced amino acid identity (%) of the selected 22 cDNA sequences from the 17 contigs.

Values shaded in blue refer to the deduced amino acid identity of fragments from the same contig, while values shaded in grey denotepercentage identities between fragments that may be parts of the same P450 gene or allele.

**Figure 4.**
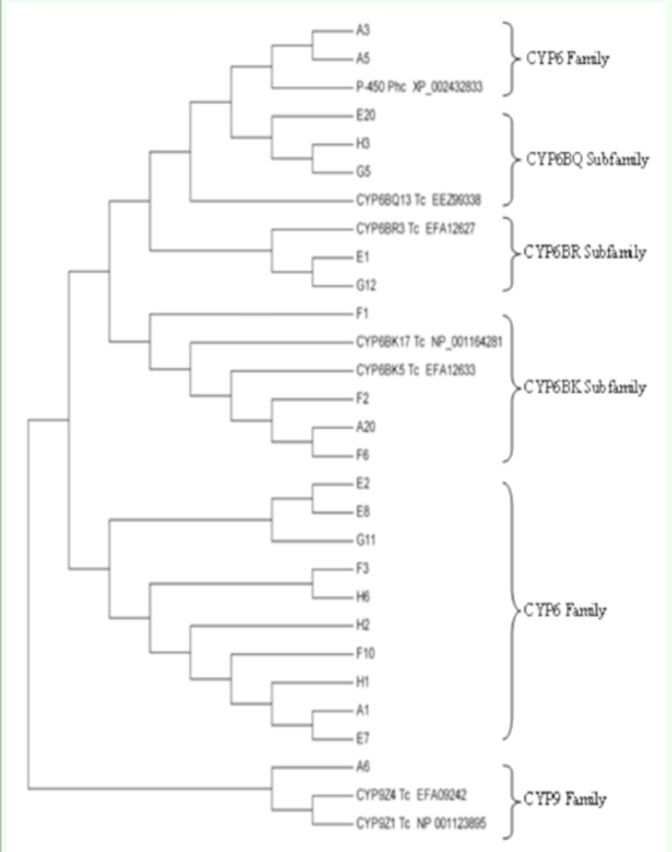
Phylogenetic relationships based on the amino acid sequence alignments of 22 novel sequences and P450 genes from CYP6 and CYP9 families. Letter designation: Phc: *Pediculus humanus corporis* ; Tc: *Tribolium castaneum* . High quality figures are available online.

Though the cDNA fragments derived from different primers or contigs usually had ≤70% deduced amino acid identity, exceptions still existed. Some deduced amino acid sequences from different groups were found to be much closer to each other. For instance, according to Table 5 and Figure 4 , the deduced amino acid identity of the corresponding regions of A1 (group I) and E7 (group II), H3 (group I) and E20 (group II) were both 97.5%, and that of H1 (group I) and E7 (group II), G5 (group II) and H3 (group I) were both 100%, which indicated that fragments H3, G5, and E20 might belong to the same P450 genes (CYP6BQ subfamily) or its allele, as well as F2, A20, and F6, which are supposed to be the parts of another P450 gene (CYP6BK subfamily) or its allele.

### Phylogenetic tree analysis

A phylogenetic tree was constructed using the neighbor-joining method ( Figure 4 ). As shown in Figure 4 , A6 was found to be less closely related to the other sequences and was classified into the CYP9 family. Fragments H3, G5, and E20 were more closely related to CYP6BQ13 than the others and shared a high deduced amino acid identity of 97% and 100%, as is shown in Table 5 , which strongly indicated that these three fragments may be different parts of the same gene from the CYP6BQ subfamily. Fragments E1 and G2 were classified into CYP6BR subfamily. A20, F1, F2, and F6 were classified into CYP6BK subfamily. Despite the high deduced amino acid identity, fragments A1, E7, and H1 were not classified into any given CYP6 subfamilies. Though sharing a low identity of amino acid, fragments E1 and G12 were still classified into the same subfamily, CYP6BR.

## Discussion

Gene cloning by PCR using degenerate primers derived from the conserved amino acid sequences of other species has been shown to be an effective method to isolate DNA sequences from target species ( Li et al. 2005 ; Yang et al. 2007 ; Huang et al. 2012 ). Though the P450 superfamily has a very diverse sequence, and the overall homology may be less than 40% even within the same family, especially in insects, several functional sequence motifs still persevered during the evolution ( Wang and Hobbs 1995 ). For example, the classic heme-binding sequence motif, FxxGxxxCxG, which is universal among all P450 enzymes. The K-helix, ExxR, and I-helix, (A/G)GxxT are also highly conserved. Degenerate primers designed based on those conserved animo acid sequences are usually utilized in the amplification of P450 genes in different species, and the results are productive ( Kasai et al. 2000 ; Jiang et al. 2008 ; Huang et al. 2012 ). In our study, five pairs of degenerate primers were designed according to those conserved motifs. Compared to the previous studies with just one pair of degenerate primer, our findings were more fruitful and reliable.

The best hits of the 100 new cDNA fragments are almost P450 genes from *T. castaneum* , which belongs to the same order as *D. helophoroides* . One possible reason for this is that the completion of the genome sequencing of *T. castaneum* makes its total number of P450 genes able to possess an overwhelming percentage in beetles. Another possible reason is that P450 genes from both *D. helophoroides* and *T. castaneum* were highly homologous due to shared ancestry. However, the real reason may not be clear until more P450 genes from beetles have been isolated. The deduced amino acid sequences of 22 cDNA fragments shared a high identity with CYP6BK17, CYP6BK5, and CYP6BQ13 from *T. castaneum* . Therefore, it is possible that there are similar P450 genes in *D. helophoroides* . According to the report of Xu et al. (2009) , CYP6BQ13v2, an allele of CYP6BQ13, played an important role in the development of *T. castaneum* . Generally speaking, as the primary structure of protein, the amino acid sequence is critical to the function of certain genes to a large extent. So, it is reasonable to suspect that there are P450 genes with similar functions as CYP6BQ13v2 in *D. helophoroides* . It is a complex and strict process to validate the function of one P450 gene; however, proper speculation can make further research smoother and more purposeful.

In addition to being a potential biological control agent, the aging of *D. helophoroides* is also of interest. However, the information about age-related P450 genes from insects is limited. But, age-related P450 genes have long been reported in both mammals and plants ( Maeda et al. 1984 ; Schenkman et al. 1989 ; Imaoka et al. 1991 ; Wauthier et al. 2007 ). Also, substantial evidence suggests that age-related changes in CYP expression occur in humans and rats ( Yun et al. 2010 ). In rats, hepatic CYP expression changes with aging ( Imaoka et al. 1991 ; Wauthier et al. 2007 ), and developmental expression of CYP isoforms has been studied extensively. According to the report of Yun (2010) , increasing age caused significant alterations in the expression of CYP isoforms. Specifically, the expression of hepatic drug-metabolizing CYP genes, such as CYP2B1, CYP2C11, and CYP3A1, peaked after puberty, and then declined during the adult stage. Due to these changes, the corresponding capacity of detoxification in the liver decreased, which led to the accumulation of toxins, both endogenous and exogenous, during aging. Consequently, all these alterations may have an adverse effect on health and may accelerate the aging process of mice. Although the mechanisms of the aging process between mice and insects are much different, the function of CYP genes in all organisms are highly conserved. Considering that CYP6 genes of insects have been shown to be an effective metabolism drug, it is very likely to play a similar role as the CYP genes in rats. This is why the CYP6 gene family was examined in our study. The expression profile of all CYP6 genes we found could be examined to see whether they are age-related or not in *D. helophoroides* .

A large number of P450 genes have been isolated and identified. However, P450 gene reports from insects are still limited. So far, more than 56,000 P450 sequences from varied organisms have been submitted to GenBank, but less than 10% of them are from insects. In beetles, the total number of P450 sequences are only 337, mostly belonging to *T. castaneum* . Except for those species in which the genome has been sequenced, the amount of research on P450 genes in other species is still low. For species without the establishment of a cDNA library and completion of genome sequencing, it is difficult to detect the overall aboundance of P450 genes. In the present study, five pairs of degenerate primers were used to amplify the P450 genes in *D. helophoroides* , and a total of 100 P450 fragments were isolated and classified, which gave an overall picture of CYP6 genes in this beetle. The findings expand the P450 gene information in beetles and add to the molecular knowlege of *D. helophoroide* .

Although the isolated P450 cDNA fragments in the current study are only 1/5 to 1/3 parts of commom full length P450 sequences, several functional critical motifs of P450s enzymes were found. In addition, the cDNA fragments amplified by PCR using degenerate primers that derived from the I-helix and heme-binding motifs could accurately reflect the authenticity of the corresponding full-length P450 genes ( Fogleman and Danielson 2001 ). Therefore, the obtained sequences and homology analysis of cDNA fragments in this study took the first step towards the exploration of a brand new area of insect P450 beyond drug resisitance in this beetle, and also provides an easy and cheap way to scan the abundance of P450 genes in certain organisms.
